# *Pseudomonas flavocrustae* sp. nov., an endophyte with plant growth promoting traits isolated from *Passiflora incarnata*

**DOI:** 10.1038/s41598-024-64349-1

**Published:** 2024-06-21

**Authors:** Luis Gabriel Cueva-Yesquén, Adilson Sartoratto, Adriana da Silva Santos, Itamar Soares de Melo, Fabiana Fantinatti-Garboggini

**Affiliations:** 1 Graduate Program in Genetics and Molecular Biology, Institute of Biology, University of Campinas, Campinas, SP Brazil; 2 Division of Microbial Resources, Research Center for Agriculture, Biological and Chemical, University of Campinas, Paulínia, SP Brazil; 3 Division of Organic and Pharmaceutical Chemical, Research Center for Agriculture, Biological and Chemical, University of Campinas, Paulínia, SP Brazil; 4 Embrapa Meio Ambiente, Rodovia SP 340 Km 127.5, CP 69, Jaguariúna, SP CEP 13820-000 Brazil

**Keywords:** Taxonomy, Microbiology, Plant symbiosis

## Abstract

A polyphasic approach was applied to characterize taxonomically a novel endophytic bacterial strain, designated as EP178^T^, which was previously isolated from *Passiflora incarnata* leaves and characterized as plant-growth promoter. The strain EP178^T^ forms Gram stain-negative and rod-shaped cells, and circular and yellow-pigmented colonies. Its growth occurs at 10–37 °C, at pH 6.0–8.0, and tolerates up to 7% (w/v) NaCl. The major cellular fatty acids found were summed feature 8 (C_18:1_ ω7c), summed feature 3 (C_16:1_ ω6c /C_16:1_ ω7c), and C_16:0_, and the predominant ubiquinone was Q-9. The phylogenetic and nucleotide-similarity analysis with 16S rRNA gene sequences showed that strain EP178^T^ belongs to *Pseudomonas* genus. The genomic-based G + C content was 65.5%. The average nucleotide identity and digital DNA-DNA hybridization values between strains EP178^T^ and the closest type strain, P. *oryzihabitans* DSM 6835^T^, were 92.6% and 52.2%, respectively. Various genes associated with plant-growth promoting mechanisms were annotated from genome sequences. Based on the phenotypic, genomic, phylogeny and chemotaxonomic data, strain EP178^T^ represents a new species of the genus *Pseudomonas*, for which the name *Pseudomonas flavocrustae* sp. nov. was proposed. The type strain is EP178^T^ (= CBMAI 2609^T^ = ICMP 24844^T^ = MUM 23.01^T^).

## Introduction

*Passiflora incarnata* is a fast-growing perennial with climbing and trailing trunks. This species is originally from South America and occurs mainly in Brazil and some other tropical regions of America, Asia, and Australia^1^. It is considered a “heavy feeder” plant, so for vegetative growth, it usually needs a balanced fertilizer with similar proportions of mainly nitrogen, phosphorus, and potassium^2^. Passionflower is recognized as an herbal medicines source by National Pharmacopoeias in France, Germany, and Switzerland, and by the Homeopathic Pharmacopoeia of the United States^3^. The wide-range therapeutic potential of P. *incarnata* can be attributed to the diverse bioactive constituents synthesized by the plant, including flavonoids, cyanogenic glycosides, and indole alkaloids^4^. This species, like other plant systems, can potentially host a diverse microbial community that plays a critical role in nutrition and health of the host.

*Pseudomonas* belongs to the phylum, recently renamed, *Pseudomonadota* and contains more than 250 species validly described (https://www.bacterio.net/), which have been reported on diverse environments^5^. Since 1984, when *Pseudomonas* was proposed as a genus, genomic backgrounds such as DNA-DNA hybridization have been used to delineate the genus boundaries^6^. House-keeping genes, including the 16S rRNA gene, were used also to organize and resolve the boundaries within the genus^7^. Actually, overall genome related index (OGRI) are the “gold-standard” genomic metrics used for delineating species in prokaryotic^8^. A recent study analyzed ten thousand *Pseudomonas* genomes and suggested that type strains represent less than half of the estimated species number^9^.

Diverse microbial lifestyles and ecological insights have been disentangled by genome sequencing and analysis. Then, genome mining can explain phenotypic traits observed through physiological and biochemical assays and expand biotechnological applications of members of the *Pseudomonas* genus^10^. Plant-growth promotion exerted by plant and soil-associated bacteria was revealed by annotating bacterial genes associated with plant nutrition and resistance^11^. A study with an ecological approach aimed to associate the endophytic *Passiflora incarnata* microbiome with the development stages of the host plant^12^. From the cultivable community, the strain EP178^T^ was isolated and provisionally identified as *Pseudomonas* sp., that in a further investigation, was characterized as a potential plant-growth promoter^13^. A polyphasic approach based on genomic, physiological, chemotaxonomic, and morphological characterizations was carried out with the strain EP178^T^ to resolve its taxonomic position within the *Pseudomonas* genus.

## Results and discussion

### Phylogenetic analysis

**Figure 1:**
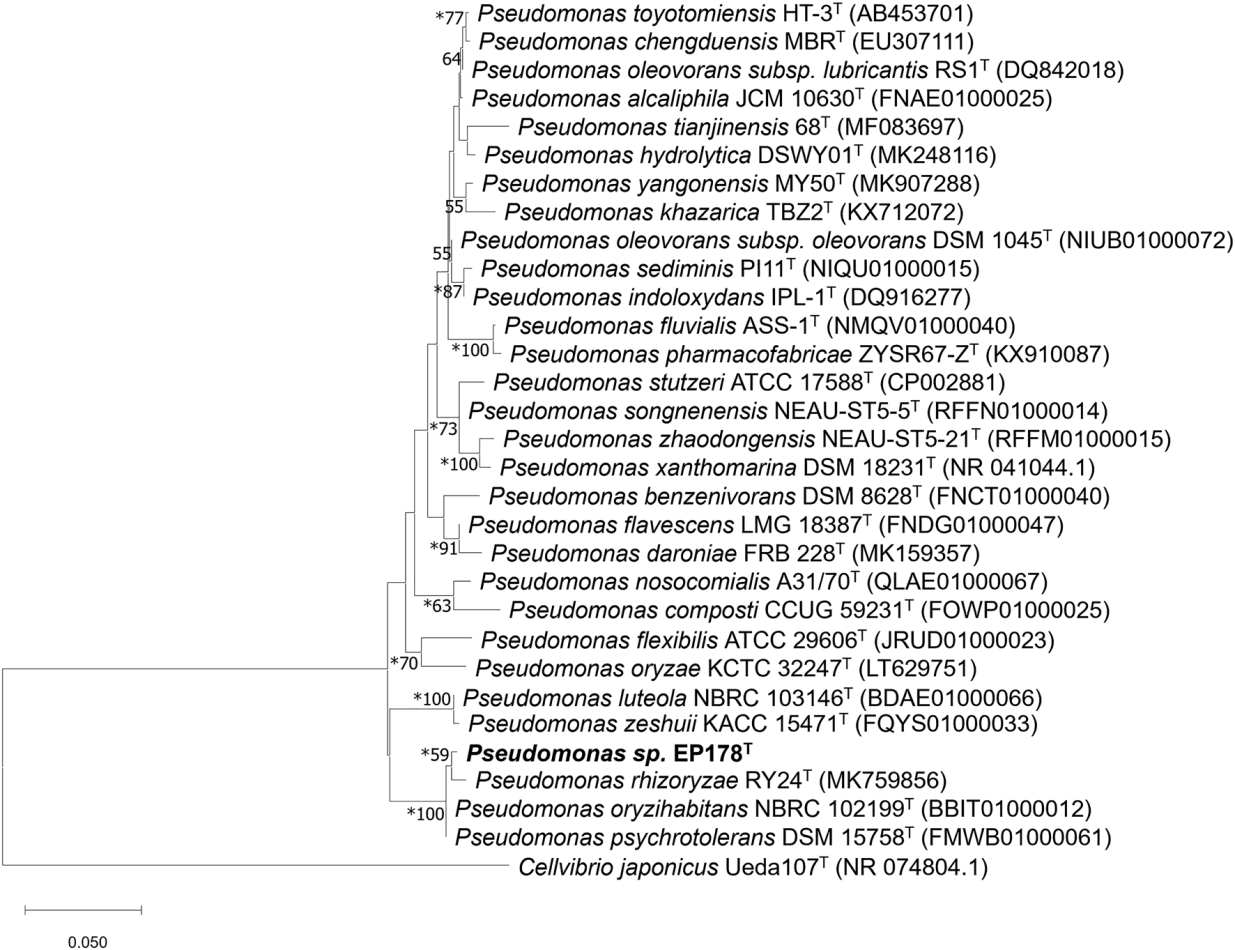
Maximum Likelihood phylogenetic tree based on 16S rRNA gene sequences of strain EP178T and its closest phylogenetically species of the *Pseudomonas* genus. This reconstruction was performed with 1406 positions and only bootstrap values ≥ 50% are shown. The nodes with asterisks are supported by three statistical methods ML, MP and NJ. *Cellvibrio japonicum* Ueda107^T^ was used as outgroup.

The Sanger sequencing of the 16S rRNA gene resulted in an assembled sequence of 1537 base pairs (bp), which had a pairwise similarity over 99% with P. *oryzihabitans* NBRC 102199^T^, P. *psychrotolerans* DSM 15758^T^ and P. *rhizoryzae* RY24^T^ in the EzBioCloud’s Identify. The values of similarity with up to thirty reference sequences are provided in Table S1. The phylogenetic tree using the ML method (Fig. 1) showed that the 16S rRNA sequence from EP178^T^ formed a distinct clade with P. *rhizoryzae*, and together with taxas P. *oryzihabitans* and P. *psychrotolerans* formed a broader cluster with a bootstrap value of 99. The three topology and sequence similarity data are inconclusive at the species level but resolutive to associate the strain EP178^T^ with the *Pseudomonas* genus. Finally, most nodes predicted are supported by three methods used (ML, MP, and NJ) in the phylogenetic reconstruction (Fig. 1).

### Genome analysis

The assembly of the EP178^T^ genome resulted in 24 sequences (scaffolds) that totalized 5,317,915 bp with coverage of 93X. The Quast evaluation calculated a N_50_ value of 390,692 bp and 65.51% of the G + C content. The CheckM analysis revealed 100% of completeness and 0.18% of contamination for the assembly, and the whole genome sequence was deposited under accession number JAPDIQ000000000. The full 16S rRNA gene sequence obtained by Sanger method was compared with the 16S rRNA sequence from WGS by pairwise alignment, resulting in 100% of identity. The NCBI pipeline predicted 4814 protein-coding genes, 71 pseudogenes, and 75 RNA genes. These latter were distributed in 7 rRNA (3 of 5S, 2 of 16S, and 23S), 58 tRNA, and 10 non-coding RNA genes (Table S2). Based on the eggNOG-mapper analysis, 4303 sequences were annotated in clusters of orthologous genes (COGs) (Table S3). Most COGs were classified as unknown (846 sequences), followed by COGs associated with amino acid metabolism and transport, transcription, inorganic ion transport and metabolism, signal transduction mechanisms, and energy production and conversion. Some of the sequences were associated with cell cycle control and defense mechanisms.

#### Taxonomic analysis

**Figure 2:**
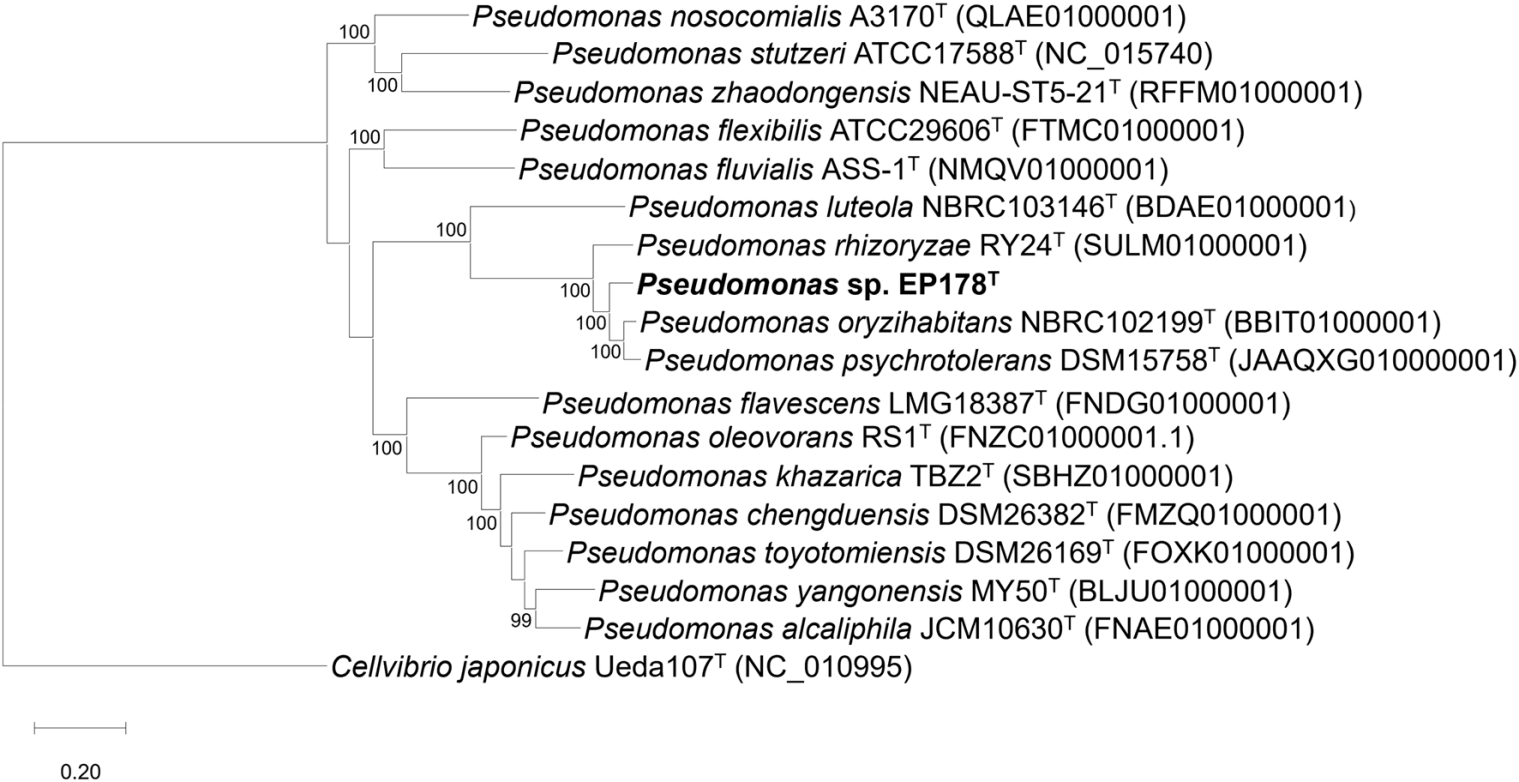
Phylogenomic tree based on *Pseudomonas* genomes included the strain EP178^T^. The evolutionary distances were calculated from the ubiquitous and informative 400 proteins. Orthologues from these proteins were detected with Diamond. The multiple alignment was inferred using MAFFT and the construction of the tree was carried out with FastTree. The genome from *Cellvibrio japonicum* Ueda107^T^ was used as outgroup.

A phylogeny analysis based on genomic sequences was conducted using the pipeline PhyloPhlAn 3.0 and showed that strain EP178^T^ forms a monophyletic group with P. *psychrotolerans*, P. *oryzihabitans* and P. *rhizoryzae* (Fig. 2), which were already reported in plant samples^14–16^.

From genomic data were calculated OGRI metrics such as ANI and dDDH, which are determinants for proposing a bacterial novel species. The threshold values for genome-based species delimitation are 95–96% for ANI and 70% for DNA-DNA reassociation^8^. For both indexes, the closest species was P. *oryzihabitans* (92.6% for ANIb and 52.2% for dDDH) (Table 1). These results confirmed that the strain belongs to an independent species from the genus *Pseudomonas*. The ANI was also estimated with aligners MUMmner and USEARCH, but the values did not exceed the threshold value with all the closest reference genomes. Specifically, the ANI values were between 84.07 and 93.71% for ANIm, and between 75.78 and 93.52% for ANIu (Table 1). Coherently, the dDDH values between the strain EP178 and all tested *Pseudomonas* members were < 52.2%.

**Table 1:** Overall genome related index (OGRI) between the strain EP178^T^ genome and the closest reference genomes.

The closest genomes	ANIb (%)	ANIm (%)	ANIu (%)	dDDH (%)	G+C (%)
*Pseudomonas oryzihabitans* DSM 6835	92.65	93.71	93.52	52.2	0.73
*Pseudomonas psychrotolerans* DSM 15758	92.62	93.60	93.45	51.5	0.19
*Pseudomonas rhizoryzae* RY24	87.15	89.36	88.50	35.3	0.68
*Pseudomonas flexibilis* ATCC 29606	74.20	84.34	76.40	21.4	0.31
*Pseudomonas stutzeri* ATCC 17588	72.71	84.13	75.50	21.0	1.2
*Pseudomonas yangonensis* MY50	73.68	84.29	75.83	21.4	2.67
*Pseudomonas toyotomiensis* DSM 26169	73.66	84.09	75.69	20.8	2.89
*Pseudomonas chengduensis* DSM 26382	73.48	84.07	75.78	20.9	3.18

#### Genomic features associated with plant-growth promotion

Plant-growth promotion (PGP) is a feature commonly attributed to microbes inhabiting the plant environment. They can support the plant development through direct and indirect mechanisms such as nutrient bioavailability or phytohormones modulation and pathogens neutralization, respectively. The genome mining of strain EP178^T^ resulted in the characterization of various PGP mechanisms by detecting accessory and essential genes associated with plant beneficial activities.

The gene (*gcd*) codifying the enzyme glucose dehydrogenase (GDH) was detected in the genome of EP178^T^. This enzyme catalyzes the glucose oxidation to gluconic acid, which can solubilize the insoluble inorganic phosphorus by acidifying the environment^17^. The cluster (*pqqB, pqqD, pqqE, pqqF, pqqL,* and *pqqM*) codifying the pyrroloquinoline quinone (PQQ) was also annotated. It is a cofactor required for phosphorus solubilization by GDH^18^. The *phoD* gene was also detected, which codifies the alkaline phosphatase. This enzyme can mineralize organic phosphorus from the soil by producing phosphatase^19^. In addition, the phosphorus uptake system was characterized by annotating the *pst* operon (*pst*S, *pst*C, *pst*A, and *pst*B). These genes are expressed when bacteria grow in phosphate-limited environments^20^. Some genes (*znu*A, *znu*B, *znu*C, *znt*B, *zur*) associated with zinc solubilization were detected. Zinc is an important micronutrient for metabolism and, therefore for host growth. The gene (*zit*B) encoding a Zn efflux exporter was also present.

Various genes (*ent*A, *ent*B, *ent*C, *ent*E, *frg*A, *yqj*H) involved in the synthesis of siderophores were annotated. Siderophores are high-affinity small molecules employed by bacteria to acquire iron from the extracellular environment. The use of siderophore-producing microbes can help to reduce Fe deficiency in plants^21^. Also, some genes (COG4774, *fep*C, *fec*B, *efe*U, and *efeO*) associated with the iron and siderophore transport were detected.

The *trp* operon (*trp*A, *trp*B, *trp*C, *trp*D, *trp*E, *trp*F, *trp*H, *trp*I, and *trp*S) was described, which is responsible for the production of tryptophan. This metabolite is an important precursor of the biosynthetic pathway of indole acetic acid (IAA). The IAA is one of the most important auxins in the modulation of the vegetative growth of plants and can be produced by bacteria^22^. Some components (*pabA* and *pabB*) of the *pab*ABC cluster were annotated, which is involved in the synthesis of anthranilate, another precursor of IAA^23^. Two genes associated with the synthesis of the enzyme aldehyde dehydrogenase were detected. This enzyme oxides indole-3-acetaldehyde in IAA^24^. Also, a gene (COG0679) encoding an auxin efflux carrier was annotated.

Several genes associated with heavy metal resistance, such as arsenic (As), copper, mercury, nickel, and cobalt, were detected in the EP178^T^ genome. For arsenic resistance were annotated genes *ars*R, *ars*B, *ars*H, *ars*C; genes *cop*A, *cop*C, *cop*D, and *cop*B for copper; genes *mer*R and *cop*Z for mercury; and genes *yoh*N and *rcn*B for nickel and cobalt.

### Morphological and biochemical characteristics

**Figure 3:**
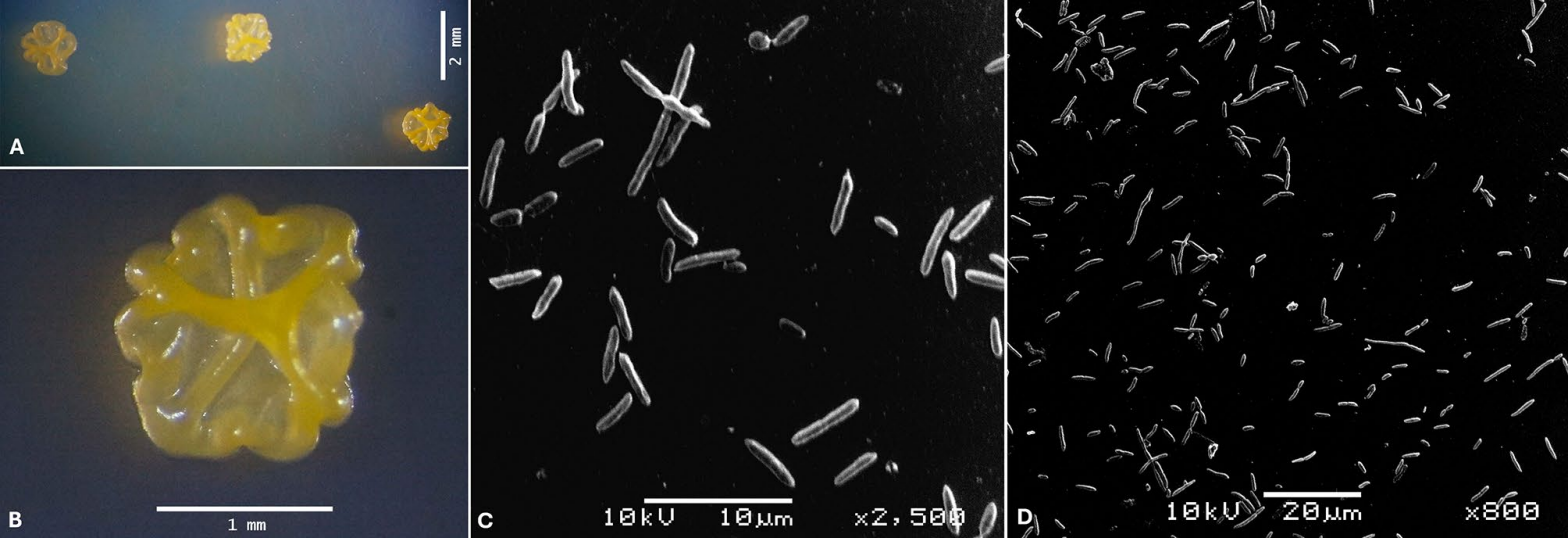
Morphologic characteristics of strain EP178^T^. (**A**) and (**B**), bacterial colonies captured by stereoscopy; (**C**) and (**D**), scanning electron micrograph of cells.

The strain EP178^T^ formed circular, with irregular borders, raised, yellow-pigmented colonies after 48 h of incubation in TSA medium at 28 °C. The cells were Gram-stain-negative, rod-shaped with approximately 0.5 µm wide and 2–5 µm long (Fig. 3). Growth was observed at 10–37 °C (optimum 30 °C), pH 6.0–8.0 (optimum 7.0), and a NaCl concentration range of 0–7% (w/v) (optimum 0%). The strain EP178^T^ is facultative anaerobe, catalase positive, and it was the only one positive for esculin hydrolysis and negative for gelatin liquefaction. The strain EP178^T^ was the only one that did not form acids from glycerol and d-lyxose but did when tested with l-fucose (Table 2). All physiological and biochemical characteristics tested in the 20NE and 50CH systems for the strain EP178^T^ are shown in Table S4.

**Table 2:** Differential physiological and biochemical features of the strain EP178^T^ with closely related type strains.

Characteristics	1	2^a^	3^b^	4^c^
Temperature range for growth (°C)	10–37	4–37	20–42	10–37
NaCl tolerance (%)	0–7	1–5	0–6.5	0–7
Hydrolysis				
Urea	−	nd	+	nd
Esculin	+	−	−	nd
Gelatin liquefaction	−	+	+	+
Acid production from				
Glycerol	−	+	+	+
d-xylose	−	+	+	+
l-fucose	+	−	−	−
d-sorbitol	−	−	+	+
d-adonithol	−	−	−	+
Starch	−	−	−	+
d-sucrose	−	−	−	+
Major respiratory quinone	Q-9	Q-9	Q-9	Q-9
G+C content (mol%)	65.5	64.9	65.1	64.25

1, EP178T; 2, P. *psychrotolerans* DSM 15758T; 3, P. *oryzihabitans* NBRC 102199T; 4, P. *rhizoryzae* RY24T.

+ positive reaction, − negative reaction, *nd* no data.

^a^Hauser et al. (2004); ^b^Kodama et al.^14^; ^c^Wang et al.^16^.

### Chemotaxonomic analysis

The chemotaxonomic features from strain EP178^T^ were coherent with typic patterns from the *Pseudomonas* genus. The major fatty acids of strain EP178^T^ were C18:1 ω7c (46.4%) and C16:1 ω7c/C16:1 ω6c (20.0%), which were also the most dominant for P. *oryzihabitans* NBRC 102199^T^. In contrast, the fatty acids C18:1 ω7c and C16:0 were the major ones for P. *psychrotolerans* DSM 15758^T^ and P. *rhizoryzae* RY24^T^. In general, minor differences were found in the fatty acid composition from EP178^T^ and the closely related reference strains (Table 3). The predominant ubiquinone for the strain in the study was Q-9. The polar lipids of the strain EP178^T^ are diphosphatidylglycerol (DPG), phosphatidylethanolamine (PE), phosphatidylglycerol (PG), two unidentified phospholipid (PL), and one unidentified lipid (L) (Fig. S1).

**Table 3:** The major fatty acids detected in strain EP178^T^ and the closest type species.

Fatty acid	1	2^a^	3^a^	4^a^
C_10:0_ 3-OH	2.9	4.1	3.8	2.8
C_12:0_	6.1	6.5	7.0	4.5
C_12:0_ 2-OH	2.8	2.7	3.4	4.2
C_12:0_ 3-OH	4.8	5.1	4.5	4.9
C_14:0_	TR	ND	ND	TR
Summed feature 3 (C_16:1_ ω6c /C_16:1_ ω7c)	20.0	18.2	17.7	16.5
C_16:0_	16.2	24.0	13.6	25.4
Summed feature 8 (C_18:1_ ω7c)	46.4	38.2	23.2	38.9
C_18:0_	TR	ND	ND	TR

1, EP178^T^; 2, P. *psychrotolerans* DSM 15758^T^; 3, P. *oryzihabitans* NBRC 102199^T^; 4, P. *rhizoryzae* RY24^T^.

*ND* no data, *TR* trace (< 1%).

^a^Wang et al.^16^.

## Conclusion

### Description of *Pseudomonas flavocrustae* sp. nov.

*Pseudomonas flavocrustae* sp. nov. (fla.vo’crus.tae. L. masc. adj. flavo-, which has a yellow color; L. gen. -crustae, with crust aspect and hard surface).

The strain EP178^T^ forms Gram stain-negative and rod-shaped cells with approximately 0.5 µm wide and 2–5 µm long. Colonies are circular, with irregular borders, raised, and yellow-pigmented after 48 h of incubation on TSA medium. The growth occurs at 10–37 °C (optimum, 30 °C), at pH 6.0–8.0 (optimum, 7.0), and tolerates up to 7% (w/v) NaCl (optimum, 0%). The strain EP178^T^ is facultative anaerobe and positive for catalase, esculin hydrolysis, and assimilation of d-glucose, l-arabinose, d-mannose, d-mannitol, d-maltose, potassium gluconate, malic acid, and trisodium citrate. The strain EP178^T^ can produce acid from d-arabinose, l-arabinose, d-ribose, d-xilose, d-galactose, d-glucose, d-fructose, d-mannose, inositol, d-mannitol, d-melibiose, d-trehalose, gentibiose, d-fucose, l-fucose, d-arabitol, and l-arabitol. The predominant ubiquinone is Q-9. The major cellular fatty acids are summed feature 8 (C_18:1_ ω7c), summed feature 3 (C_16:1_ ω6c/C_16:1_ ω7c), and C_16:0_.

The type strain is EP178^T^ (= CBMAI 2609^T^ = ICMP 24844^T^ = MUM 23.01^T^), which was isolated from P. *incarnata* leaves collected from agricultural fields of Botucatu, São Paulo, Brazil. The NBCI accession numbers for the 16S rRNA gene sequence and genome annotation of strain EP178^T^ are MG778852 and JAPDIQ000000000, respectively.

## Methods

### Isolation and culture conditions

The strain EP178 was isolated from leaf tissues in the vegetative stage of P. *incarnata* by Goulart et al.^12^. Briefly, leaves of passionfruit were collected from the Centroflora Group agricultural fields located at Botucatu, São Paulo, Brazil (23.93 S, 48.56 W) in January 2016. This isolation experiment was conducted and registered under SisGen AC29F17 (National System for the Management of Genetic Heritage and Associated Traditional Knowledge, Ministry of Environment and Climate Change, Brazil). Leaves were surface sterilized with 100% ethanol for 3 min, followed by 2% sodium hypochlorite for 2 min, 70% ethanol for 3 min, and rinsed three times with sterilized distilled water. The disinfected tissue was ground with sterilized mortars and pestles in phosphate buffered saline solution (pH 7.4). The resultant suspension was tenfold diluted up to 10^–4^, and a 100 µl aliquot was inoculated on Petri plates containing Gause’s synthetic agar^25^. Plates were incubated at 28 °C for up to 30 days. Based on the morphology, a yellow, circular, rugous colony was purified and preserved in glycerol stock at − 80 °C. This isolate was designated as EP178 and grown on trypticase soy agar (TSA, Difco) medium at 28 °C for 48 h for the next steps.

### 16S rRNA gene amplification and sequencing

The genomic DNA of strain EP178^T^ was extracted following the modified protocol of Van Soolingen et al.^26^. The 16S rRNA gene was amplified by PCR using the universal bacterial primers 10F (5-GAGTTTGATCCTGGCTCAG-3) and 1525R (5-AAGGAGGTGWTCCARCC-3) (Lane, 1991). The 25 µl PCR reaction mixture contained 0.2 mM of dNTP mix (Invitrogen), 1X buffer (Tris 20 mM, pH 8.4), 1.5 mM MgCl_2_, 0.5 µM of each primer, 1U of Taq polymerase (Invitrogen) and 10 ng of genomic DNA. The protocol of cycling consisted of an initial denaturation to 94 °C for 4 min, followed by 32 cycles of 94 °C for 1 min, 55 °C for 1 min, and 72 °C for 3 min, with a final extension to 72 °C for 5 min. The PCR product was purified using the PCR GFX™ kit (GE Healthcare Life Sciences) and amplicons were sequenced in the Genetic analyzer ABI3500XL by the Sanger method using BigDye™ Terminator v3.1 Kit (ThermoFisher Scientific) and primers 10F, 1525R, 530R (5-GWATTACCGCGGCKGCTG-3) and 968F (5-AACGCGAAGAACCTT AC-3). Reads were assembled in the software BioEdit^27^ and resultant sequences were submitted to the Identify platform from EzBioCloud (https://www.ezbiocloud.net/identify)^28^ to recover the phylogenetically closest reference sequences.

### Phylogenetic analysis

The 16S rRNA gene sequence of EP178^T^ and those recovered from EzBiocloud analysis were aligned with CLUSTAL W^29^, and the best substitution model was determined with the function Find Best DNA/Protein Models implemented in the MEGA-X software^30^. The phylogenetic reconstruction was performed using methods Maximum Likelihood (ML), Maximum Parsimony (MP), Neighbor-Joining (NJ), and the model Kimura 2-parameter with Gamma distribution (+ G) and Invariable sites (+ I). The robustness of the tree topology was evaluated with 1000 replications.

### Genome sequencing, assembly, and annotation

For the whole genome sequencing, DNA was extracted with the PowerMax Soil DNA kit (QIAGEN) following the manufacturer's instructions. The concentration of the extracted DNA was determined by fluorometry (Qubit™ 3.0, Invitrogen) and the purity was estimated by calculating the A260/A280 ratio in a spectrophotometer (NanoDrop™ 1000, Thermo Fisher Scientific). The DNA library was prepared using the Nextera XT kit and sequenced on the Illumina MiSeq sequencing platform to produce paired-end reads of 250 bp. The quality of the raw reads was accessed using the FastQC 0.11.9 (www.bioinformatics.babraham.ac.uk)^31^. The Trimmomatic v0.39 software^31^ was used to remove adapters and primers, trim low-quality ends, and filter reads with quality (Phred) less than 30. In addition, reads < 100 bp were discarded. All sequences that passed quality control were used in the genome assembly. For this, Spades v3.13^32^ was run with different values of k-mers (21–127). The assembly quality was evaluated using the Quast v5.0.2^33^ package, and the completeness and contamination were accessed with CheckM 1.1.3^34^. Gene annotation was determined by the Prokaryotic Genome Annotation Pipeline (PGPA)^35–37^. Simultaneously, gene prediction was performed using Prodigal v2.6.3^38^ and the output file was submitted to the eggNOG-mapper v2 web tool^39^. The annotation table was analyzed by searching for plant growth promotion-associated genes.

### Genome-based taxonomic analysis

To resolve the taxonomy of EP178^T^, its genome was compared with genomes of the closest *Pseudomonas* species by calculating overall genome related index (OGRI) such as Average Nucleotide Identity (ANI) and digital DNA-DNA Hybridization (dDDH). The ANI was calculated with the alignment algorithms BLAST + (ANIb), MUMmner (ANIm), and USERCH (ANIu) using the JSpecies tool (http://jspecies.ribohost.com/jspeciesws)^40^ and the ANI calculator (https://www.ezbiocloud.net/tools/ani)^41^, respectively. The dDDH was predicted using the Genome-to-Genome Distance Calculator (GGDC) (http://ggdc.dsmz.de/)^42^ using BLAST + as an alignment algorithm. To confirm that EP178^T^ belonged to the *Pseudomonas* genus, a phylogenomic approach was conducted following the PhyloPhlAn 3.0 pipeline^43^. It selects a relevant group of phylogenetic markers (n = 400) to be searched across genomes using Diamond 0.9.21^44^. The sequences of the detected genes are aligned with the MAFFT 7.487^45^ and alignments are concatenated to perform the phylogeny reconstruction with FastTree 2.1.11^46^. The phylogenomic tree was visualized and customized in the iTOL tool (http://itol.embl.de)^47^.

### Phenotypic characterization

Cell morphology of the strain EP178^T^ was investigated using scanning electron microscopy (SEM)^48,49^. Briefly, bacteria will be cultured in TSB medium overnight at 28 °C, the culture must be washed with PBS (pH 7.4) and fixed in 0.1 M cacodylate ((CH_3_)_2_AsO_2_Na · 3H_2_O) buffer containing and 2% paraformaldehyde. Cells will be post-fixed in 1% osmium tetroxide, dehydrated in graded ethanol series (20, 40, 60, 80, 95, and 100% ethanol) and critical point dried with CO_2_. The strain EP178^T^ was grown on TSA medium at 28 °C for 48 h to characterize the colony morphology. A single colony was stained by standard Gram procedure. Catalase activity was determined by adding a 3% (v/v) H_2_O_2_ solution and the production of bubbles was considered as a positive reaction. Also, it was characterized the oxygen requirements from by testing the growth in thioglycolate broth. The growth of strain EP178 was evaluated at different temperatures (4, 10, 15, 20, 25, 28, 30, 37, 40, 45, and 50 °C) on trypticase soy broth (TSB, Difco) medium. The pH range (4.0 to 10.0, with intervals of 1.0 pH unit) and NaCl tolerance (1 to 10% NaCl, w/v, with intervals of 1%) were evaluated on the TSB medium. The pH was adjusted before sterilization with 0.1 M citric acid/0.1 M sodium citrate (pH 4.0–5.0), 0.1 M KH_2_PO_4_/0.1 M NaOH (pH 6.0–8.0), and 0.1 M NaHCO_3_/0.1 M Na_2_CO_3_ (pH 9.0–10.0). The growth was determined by obtaining a reading of OD_600nm_ ≥ 0.1. Other physiological and biochemical properties, such as substrate utilization, enzyme activities, and acid production from carbohydrates, were determined using the kits API 20NE and API 50CH (bioMérieux) following the manufacturer’s instructions. All strips were read after 24 h and 48 h.

### Chemotaxonomic characterization

The fatty acid patterns were determined by extracting and analyzing the fatty acid methyl esters (FAMEs). Biomass was saponified, and the cellular fatty acids were extracted, methylated and quantified by gas chromatography (Agilent 6850 GC). The FAMEs were identified with the TSBA6 database 6.10 of the standard Sherlock Microbial Identification (MIDI) system.

Respiratory quinones and polar lipids were characterized following the protocol of Minnikin et al.^50^. Briefly, ubiquinones are extracted from 50 mg dry cell biomass with methanol saline (0.3% NaCl: MetOH, 1:10 v/v) and petroleum ether. The extract was separated by running the sample on Preparative Layer Chromatography (PLC) using Silica Gel 60 (F_254_, 1 mm) and petroleum ether: acetone (95:5 v/v) as the mobile phase. The separation profiling was revealed under UV light (254 nm) and the band with R_F_ 0.8–0.9 was recovered with diethyl ether, which was evaporated under gaseous nitrogen continuous flow. The purified ubiquinones were analyzed by High-Performance Liquid Chromatography (HPLC) using a C18 column (150 × 2.1 mm, 5 µm) at 30 °C, with acetonitrile: tetrahydrofuran (70:30, v/v) as mobile phase at 270 nm. For the polar lipid extraction, the same cell biomass with chloroform–methanol-0.3% saline solution (9:10:3, v/v) was performed by two-dimensional Thin Layer Chromatography (TLC). The stationary phase was Silica gel 60 (F254, Aluminum sheets) and solvents for the first and the second running were chloroform–methanol–water (64:27:5, v/v) and chloroform–acetic acid–methanol–water (80:18:12: 5, v/v), respectively. Total lipids were revealed with 10% ethanolic molybdophosphoric acid, amino lipids with 0.2% ninhydrin, and glycolipids with α-naphthol–sulfuric acid.

### Supplementary Information

Supplementary Information.

## Data Availability

The strain EP178^T^ is available from the International Collection of Microorganisms from Plants (ICMP 24844^T^), the Micoteca da Universidade do Minho (MUM 23.01^T^) and the Brazilian Collection of Environmental and Industrial Microorganisms (CBMAI 2609^T^). The 16S rRNA gene sequence from EP178^T^ is deposited under accession number MG778852. The BioSample and BioProject accession numbers are SAMN31438098 and PRJNA430160, respectively. The assembled genome sequences of strain EP178^T^ are deposited under accession number JAPDIQ000000000.
