# Supplementary material for: Pseudomonas flavocrustae sp. nov., an endophyte with plant growth promoting traits isolated from Passiflora incarnata
Source: Sci Rep. 2024 Jun 21;14:14285. doi: 10.1038/s41598-024-64349-1 (PMC11190252; doi:10.1038/s41598-024-64349-1)
Supplement: Supplementary file 1 — Supplementary Information. [file 41598_2024_64349_MOESM1_ESM.pdf]

Supplementary Information

Pseudomonas flavocrustae* sp. nov., an endophyte with plant growth promoting traits isolated from *Passiflora incarnata.

Luis Gabriel Cueva-Yesquén[1,2]*, Adilson Sartoratto[3], Adriana da Silva Santos[3], Itamar Soares de Melo[4], Fabiana Fantinatti-Garboggini[1,2*]

[1]Graduate Program in Genetics and Molecular Biology, Institute of Biology, University of Campinas (UNICAMP), Campinas, SP, Brazil

[2]Division of Microbial Resources, Research Center for Agriculture, Biological and Chemical, University of Campinas, Paulínia, SP, Brazil

[3]Division of Organic and Pharmaceutical Chemical, Research Center for Agriculture, Biological and Chemical, University of Campinas, Paulínia, SP, Brazil

[4]Embrapa Meio Ambiente, Rodovia SP 340 Km 127.5, CP 69, CEP 13820-000, Jaguariúna, SP, Brazil

*luisg_cueva@yahoo.es

*fabianaf@cpqba.unicamp.br

Table S1. Nucleotide similarity values between 16S rRNA gene sequences from strain EP178^{T} and closely related type strains. Data was obtained from the Identify server at EzBioCloud.

**Rank**	**Name**	**Strain**	**Accession**	**Similarity (%)**
1	*Pseudomonas oryzihabitans*	NBRC 102199	BBIT01000012	99,6
2	*Pseudomonas psychrotolerans*	DSM 15758	FMWB01000061	99,6
3	*Pseudomonas rhizoryzae*	RY24	MK759856	99,3
4	*Pseudomonas tianjinensis*	68	MF083697	96,8
5	*Pseudomonas stutzeri*	ATCC 17588	CP002881	96,6
6	*Pseudomonas toyotomiensis*	HT-3	AB453701	96,4
7	*Pseudomonas chengduensis*	MBR	EU307111	96,4
8	*Pseudomonas yangonensis*	MY50	MK907288	96,4
9	*Pseudomonas oleovorans subsp. Lubricantis*	RS1	DQ842018	96,4
10	*Pseudomonas flavescens*	LMG 18387	FNDG01000047	96,3
11	*Pseudomonas alcaliphila*	JCM 10630	FNAE01000025	96,3
12	*Pseudomonas zhaodongensis*	NEAU-ST5-21	RFFM01000015	96,3
13	*Pseudomonas fluvialis*	ASS-1	NMQV01000040	96,3
14	*Pseudomonas khazarica*	TBZ2	KX712072	96,3
15	*Azotobacter chroococcum subsp. Chroococcum*	IAM 12666	AB175653	96,3
16	*Pseudomonas oleovorans subsp. Oleovorans*	DSM 1045	NIUB01000072	96,2
17	*Pseudomonas luteola*	NBRC 103146	BDAE01000066	96,2
18	*Pseudomonas flexibilis*	ATCC 29606	JRUD01000023	96,2
19	*Pseudomonas nosocomialis*	A31/70	QLAE01000067	96,2
20	*Pseudomonas pharmacofabricae*	ZYSR67-Z	KX910087	96,1
21	*Pseudomonas zeshuii*	KACC 15471	FQYS01000033	96,1
22	*Pseudomonas benzenivorans*	DSM 8628	FNCT01000040	96,0
23	*Pseudomonas hydrolytica*	DSWY01	MK248116	96,0
24	*Pseudomonas daroniae*	FRB 228	MK159357	96,0
25	*Pseudomonas guguanensis*	JCM 18416	FNJJ01000024	95,9
26	*Pseudomonas turukhanskensis*	IB1.1	KP306892	95,9
27	*Pseudomonas xanthomarina*	DSM 18231	jgi.1021496	95,9
28	*Pseudomonas guariconensis*	LMG 27394	FMYX01000029	95,9
29	*Pseudomonas alcaligenes*	NBRC 14159	BATI01000076	95,8
30	*Pseudomonas composti*	CCUG 59231	FOWP01000025	95,8

Table S2. Genomic features annotated for the strain EP178^T.

Features	Number
Genes (total)	4,960
CDSs (total)	4,885
Genes (coding)	4,814
CDSs (with protein)	4,814
Genes (RNA)	75
rRNAs	3, 2, 2 (5S, 16S, 23S)
Complete rRNAs	1, 1 (5S, 23S)
Partial rRNAs	2, 2, 1 (5S, 16S, 23S)
tRNAs	58
ncRNAs	10
Pseudo Genes (total)	71
CDSs (without protein)	71
Pseudo Genes (ambiguous residues)	0 of 71
Pseudo Genes (frameshifted)	20 of 71
Pseudo Genes (incomplete)	50 of 71
Pseudo Genes (internal stop)	25 of 71
Pseudo Genes (multiple problems)	20 of 71

Table S3. Clusters of orthologous genes (COGs) identified in the strain EP178^T genome based on the eggNOG-mapper annotation.

**COGs**	**Number**
Chromatin Structure and dynamics and Secondary Structure	3
Energy production and conversion	301
Cell cycle control and mitosis	54
Amino Acid metabolism and transport	447
Nucleotide transport and metabolism	121
Carbohydrate transport and metabolism	219
Coenzyme transport and metabolism	156
Lipid transport and metabolism	183
Translation, ribosomal structure and biogenesis	214
Transcription	395
Replication, recombination and repair	175
Cell wall/membrane/envelope biogenesis	244
Cell motility	149
Posttranslational modification, protein turnover, chaperones	121
Inorganic ion transport and metabolism	311
Secondary metabolites biosynthesis, transport and catabolism	54
Function unknown	846
Signal transduction mechanisms	210
Intracellular trafficking, secretion, and vesicular transport	58
Defense mechanisms	42
TOTAL	4303

Table S4. Biochemical and physiological profiling for the strain EP178^T and closely related type strains.

Characteristics	1	2	3	4
Nitrate	-	nd	-	nd
L-tryptophane	-	-	-	nd
D-glucose	-	nd	nd	nd
L-arginine	-	nd	-	nd
Urea	-	nd	+	nd
Esculin	+	-	-	nd
Gelatin	-	+	+	+
4-nitrophenyl-BD-galactopyranoside	-	-	-	nd
**Assimilation**				
D-glucose	+	+	+	+
L-arabinose	+	+	+	nd
D-mannose	+	+	+	+
D-mannitol	+	+	+	+
N-acetyl-glucosamine	-	-	-	-
D-maltose	+	+	+	nd
Potassium gluconate	+	+	+	nd
Capric acid	-	nd	nd	nd
Adipic acid	-	-	nd	nd
Malic acid	+	+	nd	+
Trisodium citrate	+	nd	+	nd
Phenylacetic acid	-	-	-	-
**Acid from:**				
Glycerol	-	+	+	+
Erythritol	-	-	-	-
D-arabinose	+	+	+	+
L-arabinose	+	+	+	+
D-ribose	+	+	-	+
D-xylose	+	+	+	+
L-xylose	-	-	-	-
D-adonithol	-	-	-	+
Metil-B-D-xiloside	-	-	-	-
D-galactose	+	+	+	+
D-glicose	+	+	+	+
D-fructose	+	+	+	+
D-mannose	+	+	+	+
L-sorbose	-	-	-	-
L-Rhamnose	-	-	-	-
Dulcitol	-	-	-	-
Inositol	+	+	+	+
D-mannitol	+	+	+	+
D-sorbitol	-	-	+	+

Metil-alfa-D-mannoside	-	-	-	-
Metil-alfa-D-glucoside	-	-	-	-
N-acetyl-glucosamine	-	-	-	-
Amygdalin	-	-	-	-
Arbutin	-	-	-	-
Esculine	-	nd	nd	nd
Salicin	-	-	-	-
D-cellobiose	-	-	-	-
D-maltose	-	-	-	-
D-lactose	-	-	-	nd
D-melibiose	+	+	+	+
D-sucrose	-	-	-	+
D-trehalose	+	+	+	+
Inulin	-	-	-	-
D-melizitose	-	-	-	-
D-raffinose	-	-	-	-
Starch	-	-	-	+
Glycogen	-	nd	nd	-
Xylitol	-	-	-	-
Gentibiose	+	nd	nd	nd
D-turanose	-	nd	nd	+
D-lyxose	-	+	+	+
D-tagatose	-	-	-	-
D-fucose	+	+	+	+
L-fucose	+	-	-	-
D-arabitol	+	+	nd	nd
L-arabitol	-	-	-	-
Gluconate	-	nd	nd	nd
2 Keto-gluconate	-	-	-	-
5 Keto-gluconate	-	-	-	-

1, EP178^T; 2, P. *psychrotolerans* DSM 15758^T; 3, P. *oryzihabitans* NBRC 102199^T; 4, P. *rhizoryzae* RY24^T.
a, Hauser et al., 2004; b, Kodama et al., 1985; c, Wang et al., 2020.
+, positive reaction; −, negative reaction; nd, no data

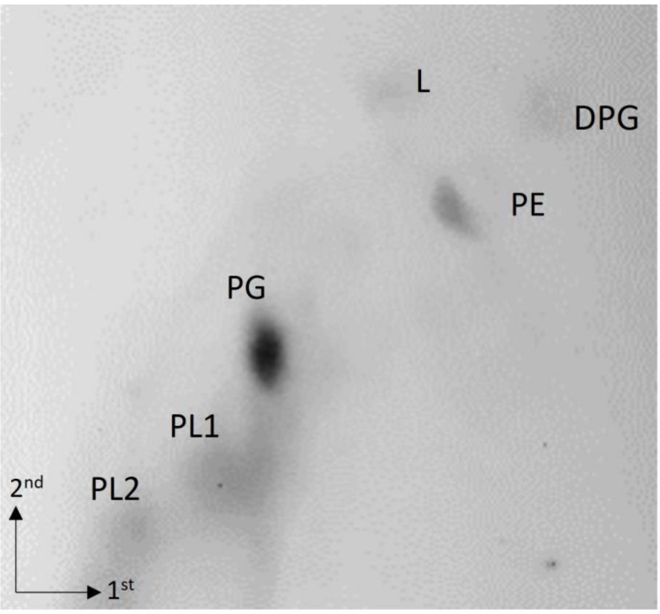

Figure S1. Polar lipid profile by two-dimensional thin-layer chromatography of the strain EP178^T. The positions of DPG, diphosphatidylglycerol; PE, phosphatidylethanolamine; L, unknown lipid; PG, phosphatidylglycerol; PL1 and PL2, unknown phospholipids
